# Ethyl (2*E*)-2-(2*H*-1,3-benzodioxol-5-yl­methyl­idene)-4-chloro-3-oxobutano­ate

**DOI:** 10.1107/S1600536811011780

**Published:** 2011-04-07

**Authors:** Julio Zukerman-Schpector, Siti Nadiah Abd Salim, Paulo J. S. Moran, Bruno R. S. de Paula, J. Augusto R. Rodrigues, Edward R. T. Tiekink

**Affiliations:** a Department of Chemistry, Universidade Federal de São Carlos, 13565-905 São Carlos, SP, Brazil; b Department of Chemistry, University of Malaya, 50603 Kuala Lumpur, Malaysia; c Instituto de Química, Universidade Estadual de Campinas, CP 6154, 13083-970 Campinas, SP, Brazil

## Abstract

In the title compound, C_14_H_13_ClO_5_, the five-membered ring is in an envelope conformation with the methyl­ene C-atom being the flap. The conformation about the C=C double bond [1.341 (2) Å] is *E*. The chloro­propan-2-one residue is approximately orthogonal to the remaining mol­ecule [dihedral angle = 88.03 (6)°]. In the crystal, the mol­ecules associate *via* C—H⋯O inter­actions, involving both carbonyl-O atoms, giving rise to an undulating two-dimensional array in the *ac* plane.

## Related literature

For background to the study, see: Rodrigues *et al.* (2004 ▶). For ring conformational analysis, see: Cremer & Pople (1975 ▶).
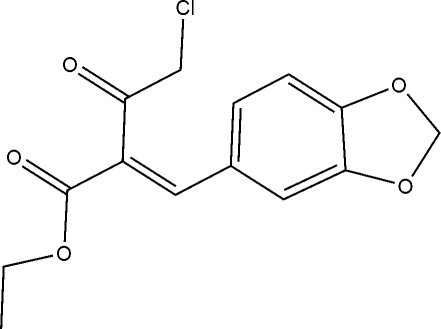


## Experimental

### 

#### Crystal data

C_14_H_13_ClO_5_
*M* *_r_* = 296.69
Orthorhombic, 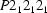

*a* = 4.8042 (1) Å
*b* = 14.9134 (4) Å
*c* = 18.4793 (5) Å
*V* = 1323.99 (6) Å^3^
*Z* = 4
Mo *K*α radiation
μ = 0.31 mm^−1^
*T* = 100 K
0.28 × 0.07 × 0.06 mm

#### Data collection

Bruker SMART APEX diffractometer
Absorption correction: multi-scan (*SADABS*; Sheldrick, 1996 ▶) *T*_min_ = 0.636, *T*_max_ = 0.746
13030 measured reflections
3200 independent reflections
2915 reflections with *I* > 2σ(*I*)
*R*_int_ = 0.041

#### Refinement

*R*[*F* ^2^ > 2σ(*F* ^2^)] = 0.031
*wR*(*F* ^2^) = 0.073
*S* = 1.05
3200 reflections
182 parameters
H-atom parameters constrained
Δρ_max_ = 0.28 e Å^−3^
Δρ_min_ = −0.18 e Å^−3^
Absolute structure: Flack (1983 ▶), 1312 Friedel pairs
Flack parameter: −0.03 (5)

### 

Data collection: *APEX2* (Bruker, 2009 ▶); cell refinement: *SAINT* (Bruker, 2009 ▶); data reduction: *SAINT*; program(s) used to solve structure: *SIR97* (Altomare *et al.*, 1999 ▶); program(s) used to refine structure: *SHELXL97* (Sheldrick, 2008 ▶); molecular graphics: *ORTEP-3* (Farrugia, 1997 ▶) and *DIAMOND* (Brandenburg, 2006 ▶); software used to prepare material for publication: *Marvin­Sketch* (Chemaxon, 2010 ▶) and *publCIF* (Westrip, 2010 ▶).

## Supplementary Material

Crystal structure: contains datablocks global, I. DOI: 10.1107/S1600536811011780/hg5017sup1.cif

Structure factors: contains datablocks I. DOI: 10.1107/S1600536811011780/hg5017Isup2.hkl

Additional supplementary materials:  crystallographic information; 3D view; checkCIF report

## Figures and Tables

**Table 1:** Hydrogen-bond geometry (Å, °)

*D*—H⋯*A*	*D*—H	H⋯*A*	*D*⋯*A*	*D*—H⋯*A*
C1—H1a⋯O4^i^	0.97	2.50	3.348 (2)	146
C11—H11b⋯O3^ii^	0.97	2.36	3.1585 (18)	139

Symmetry codes: (i) 
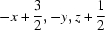
; (ii) 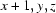.

**Fig. 1.**
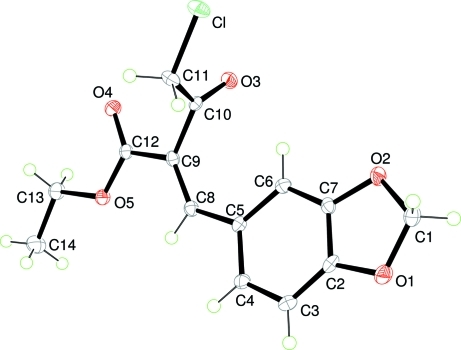
The molecular structure of compound (I) showing the atom-labelling scheme and displacement ellipsoids at the 50% probability level.

**Fig. 2.**
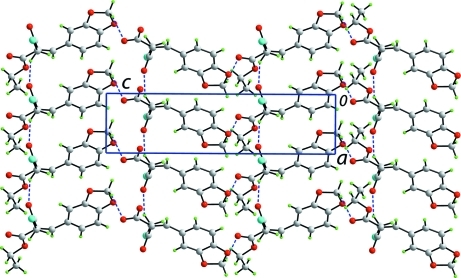
view of the supramolecular layer in (I) mediated by C—H···O interactions (blue dashed lines).

**Fig. 3.**
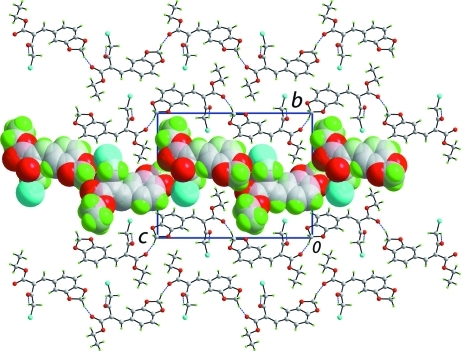
A view in projection down the a axis of the unit-cell contents for (I). The view highlights the undulating topology for the layer shown in Fig. 2, with one layer emphasized in space flling mode. The C—H···O interactions are shown as blue dashed lines.

## supplementary crystallographic information

### Comment

As part of the continuing interest in the bio-reduction of α-haloketones and enones (Rodrigues *et al.*, 2004) the title compound, ethyl (2*E*)-2-(2*H*-1,3-benzodioxol-5-ylmethylidene)-4- chloro-3-oxobutanoate, (I), was synthesized by means of a Knoevenagel condensation reaction between ethyl 4-chloroacetoacetate and piperonal, affording a mixture of *E* and *Z* isomers that were separated by column chromatography (hexane/ethyl acetate, gradient from pure hexane to 95% hexane/5% ethyl acetate).

The conformation of the five-membered ring in (I) is an envelope with the methylene-C1 atom being the flap. This is quantified by the ring puckering parameters Q(2) = 0.1016 (14) Å and φ_2_ = 38.4 (8) ° (Cremer & Pople, 1975). The conformation about the C8═C9 double bond [1.341 (2) Å] is *E*. With the exception of the chloropropan-2-one residue which projects to one side of the molecule, (I) is approximately flat. The major twist in the molecule occurs around the C5—C8 bond as seen in the value of the C4—C5—C8—C9 torsion angle of 165.21 (16) °. The carbonyl and chlorido atoms of the chloropropan-2-one residue lie in a plane with the C_3_ backbone. This plane is orthogonal to the main residue with the dihedral angle between the least-squares plane through the O3,C9—C11,Cl1 atoms and that through the C8,C9,C12 atoms being 88.03 (6) °. Both heteroatoms project away from the main residue.

The two most prominent interactions operating in the crystal structure are of the type C—H···O, Table 1. The shortest interaction occurs between symmetry related carbonyl-O and methylene-H atoms of the chloropropan-2-one residues leading to a chain along the *a* direction. The other C—H···O interaction occurs between the ester-carbonyl-O and the methylene-H of the benzodioxole residue. Overall, the resulting supramolecular architecture is a 2-D array in the *ac* plane, Fig. 2. The topology is undulating as seen from Fig. 3.

### Experimental

The title compound, (I), was prepared by means of a Knoevenagel condensation reaction between ethyl 4-chloroacetoacetate and piperonal. Morpholine (17.4 mg, 0.2 mmol), glacial acetic acid (12.0 mg, 0.2 mmol) and piperonal (300 mg, 2 mmol) were added to [Bmim][NTf_2_] (1 ml) in a 10 ml round-bottom flask. The mixture was stirred for 10 min., and then ethyl 4-chloroacetoacetate (395 mg, 2.4 mmol) and 4 Å molecular sieves (360 mg) were added to reaction mixture. After 1.5 h, the ionic liquid layer was extracted with diethyl ether (3 *x* 5 ml), the ether was evaporated to afford a mixture of *E* and *Z* isomers that were separated by column chromatography (hexane/ethyl acetate, gradient from pure hexane to 95% hexane/5% ethyl acetate). The crystallized isomer, obtained by slow evaporation from a dichloromethane/hexane mixture, was shown by crystallography to be the *E* isomer; *M*.pt. 367.1–367.4 K.

### Refinement

Carbon-bound H-atoms were placed in calculated positions (C—H 0.93 to 0.97 Å) and were included in the refinement in the riding model approximation, with *U_iso_*(H) = 1.2*U_eq_*(C) and 1.*5U*_eq_(methyl-C).

### Crystal data

**Table:**

C_14_H_13_ClO_5_	*F*(000) = 616
*M**_r_* = 296.69	*D*_x_ = 1.488 Mg m^−^^3^
Orthorhombic, *P*2_1_2_1_2_1_	Mo *K*α radiation, λ = 0.71073 Å
Hall symbol: P 2ac 2ab	Cell parameters from 4024 reflections
*a* = 4.8042 (1) Å	θ = 2.6–28.6°
*b* = 14.9134 (4) Å	µ = 0.31 mm^−^^1^
*c* = 18.4793 (5) Å	*T* = 100 K
*V* = 1323.99 (6) Å^3^	Needle, colourless
*Z* = 4	0.28 × 0.07 × 0.06 mm

### Data collection

**Table:**

Bruker SMART APEX diffractometer	3200 independent reflections
Radiation source: fine-focus sealed tube	2915 reflections with *I* > 2σ(*I*)
graphite	*R*_int_ = 0.041
ω scan	θ_max_ = 28.0°, θ_min_ = 1.8°
Absorption correction: multi-scan (*SADABS*; Sheldrick, 1996)	*h* = −6→6
*T*_min_ = 0.636, *T*_max_ = 0.746	*k* = −19→19
13030 measured reflections	*l* = −24→24

### Refinement

**Table:**

Refinement on *F*^2^	Secondary atom site location: difference Fourier map
Least-squares matrix: full	Hydrogen site location: inferred from neighbouring sites
*R*[*F*^2^ > 2σ(*F*^2^)] = 0.031	H-atom parameters constrained
*wR*(*F*^2^) = 0.073	*w* = 1/[σ^2^(*F*_o_^2^) + (0.0415*P*)^2^] where *P* = (*F*_o_^2^ + 2*F*_c_^2^)/3
*S* = 1.05	(Δ/σ)_max_ < 0.001
3200 reflections	Δρ_max_ = 0.28 e Å^−^^3^
182 parameters	Δρ_min_ = −0.18 e Å^−^^3^
0 restraints	Absolute structure: Flack (1983), 1312 Friedel pairs
Primary atom site location: structure-invariant direct methods	Flack parameter: −0.03 (5)

### Special details

**Table:**

Geometry. All s.u.'s (except the s.u. in the dihedral angle between two l.s. planes) are estimated using the full covariance matrix. The cell s.u.'s are taken into account individually in the estimation of s.u.'s in distances, angles and torsion angles; correlations between s.u.'s in cell parameters are only used when they are defined by crystal symmetry. An approximate (isotropic) treatment of cell s.u.'s is used for estimating s.u.'s involving l.s. planes.
Refinement. Refinement of *F*^2^ against ALL reflections. The weighted *R*-factor *wR* and goodness of fit *S* are based on *F*^2^, conventional *R*-factors *R* are based on *F*, with *F* set to zero for negative *F*^2^. The threshold expression of *F*^2^ > σ(*F*^2^) is used only for calculating *R*-factors(gt) *etc*. and is not relevant to the choice of reflections for refinement. *R*-factors based on *F*^2^ are statistically about twice as large as those based on *F*, and *R*- factors based on ALL data will be even larger.

### Fractional atomic coordinates and isotropic or equivalent isotropic displacement parameters (Å^2^)

**Table:**

	*x*	*y*	*z*	*U*_iso_*/*U*_eq_	
Cl	0.61259 (8)	−0.13689 (2)	0.67747 (2)	0.02387 (11)	
O1	0.3303 (2)	0.08014 (8)	1.03191 (6)	0.0210 (3)	
O2	0.1425 (2)	0.01153 (7)	0.93013 (5)	0.0176 (2)	
O3	0.3855 (2)	0.04539 (7)	0.66362 (5)	0.0184 (2)	
O4	0.8831 (2)	0.13250 (7)	0.57678 (5)	0.0175 (2)	
O5	1.0712 (2)	0.23928 (7)	0.64786 (5)	0.0166 (2)	
C1	0.1462 (4)	0.00951 (11)	1.00813 (8)	0.0184 (3)	
H1A	0.2128	−0.0482	1.0251	0.022*	
H1B	−0.0397	0.0191	1.0271	0.022*	
C2	0.4641 (3)	0.10950 (10)	0.97104 (8)	0.0146 (3)	
C3	0.6715 (3)	0.17232 (10)	0.96631 (8)	0.0167 (3)	
H3	0.7441	0.2001	1.0072	0.020*	
C4	0.7684 (3)	0.19244 (10)	0.89674 (8)	0.0154 (3)	
H4	0.9067	0.2356	0.8914	0.018*	
C5	0.6645 (3)	0.14994 (9)	0.83495 (8)	0.0132 (3)	
C6	0.4459 (3)	0.08662 (10)	0.84196 (8)	0.0148 (3)	
H6	0.3688	0.0587	0.8017	0.018*	
C7	0.3532 (3)	0.06826 (9)	0.91020 (8)	0.0140 (3)	
C8	0.7904 (3)	0.17377 (10)	0.76594 (8)	0.0144 (3)	
H8	0.8986	0.2255	0.7666	0.017*	
C9	0.7744 (3)	0.13267 (10)	0.70152 (8)	0.0136 (3)	
C10	0.6222 (3)	0.04657 (10)	0.68599 (7)	0.0135 (3)	
C11	0.7940 (3)	−0.03659 (10)	0.69812 (9)	0.0177 (3)	
H11A	0.8527	−0.0384	0.7483	0.021*	
H11B	0.9600	−0.0333	0.6684	0.021*	
C12	0.9143 (3)	0.16725 (10)	0.63571 (8)	0.0136 (3)	
C13	1.2296 (4)	0.27097 (11)	0.58593 (9)	0.0214 (4)	
H13A	1.1049	0.2886	0.5472	0.026*	
H13B	1.3509	0.2239	0.5681	0.026*	
C14	1.3985 (4)	0.34985 (11)	0.61063 (9)	0.0251 (4)	
H14A	1.2761	0.3969	0.6263	0.038*	
H14B	1.5112	0.3711	0.5713	0.038*	
H14C	1.5162	0.3321	0.6501	0.038*	

### Atomic displacement parameters (Å^2^)

**Table:**

	*U*^11^	*U*^22^	*U*^33^	*U*^12^	*U*^13^	*U*^23^
Cl	0.0263 (2)	0.01483 (17)	0.0305 (2)	−0.00619 (17)	−0.00241 (18)	−0.00049 (16)
O1	0.0265 (6)	0.0228 (6)	0.0136 (5)	−0.0044 (5)	0.0053 (5)	0.0004 (5)
O2	0.0181 (6)	0.0201 (5)	0.0146 (5)	−0.0027 (5)	0.0027 (5)	0.0025 (4)
O3	0.0143 (5)	0.0232 (5)	0.0177 (5)	−0.0013 (5)	−0.0021 (5)	−0.0022 (4)
O4	0.0194 (5)	0.0206 (5)	0.0126 (5)	−0.0016 (5)	0.0009 (5)	−0.0006 (4)
O5	0.0197 (6)	0.0172 (5)	0.0128 (5)	−0.0048 (5)	0.0045 (4)	0.0007 (4)
C1	0.0193 (8)	0.0211 (8)	0.0147 (7)	0.0004 (7)	0.0024 (6)	0.0040 (6)
C2	0.0179 (7)	0.0152 (7)	0.0108 (7)	0.0060 (6)	0.0035 (6)	0.0005 (6)
C3	0.0208 (8)	0.0169 (7)	0.0125 (7)	0.0019 (6)	−0.0018 (6)	−0.0029 (6)
C4	0.0171 (7)	0.0112 (7)	0.0179 (8)	0.0002 (6)	0.0016 (6)	−0.0013 (6)
C5	0.0149 (7)	0.0121 (7)	0.0125 (7)	0.0025 (6)	0.0010 (6)	0.0008 (5)
C6	0.0158 (7)	0.0150 (7)	0.0137 (7)	0.0007 (6)	−0.0012 (6)	−0.0013 (6)
C7	0.0108 (7)	0.0120 (6)	0.0192 (7)	0.0013 (6)	0.0009 (6)	0.0006 (6)
C8	0.0134 (7)	0.0126 (7)	0.0172 (8)	0.0001 (6)	0.0009 (6)	0.0012 (6)
C9	0.0115 (6)	0.0148 (7)	0.0144 (7)	0.0007 (6)	−0.0010 (5)	0.0021 (6)
C10	0.0156 (7)	0.0178 (7)	0.0071 (6)	−0.0018 (6)	0.0027 (6)	−0.0010 (5)
C11	0.0166 (7)	0.0141 (7)	0.0223 (8)	−0.0041 (6)	−0.0032 (6)	0.0000 (6)
C12	0.0113 (7)	0.0141 (7)	0.0154 (7)	0.0025 (6)	−0.0004 (6)	0.0003 (6)
C13	0.0252 (9)	0.0238 (8)	0.0153 (8)	−0.0066 (7)	0.0058 (7)	0.0015 (7)
C14	0.0304 (9)	0.0235 (8)	0.0214 (8)	−0.0107 (8)	0.0035 (8)	0.0023 (7)

### Geometric parameters (Å, °)

**Table:**

Cl—C11	1.7728 (15)	C5—C6	1.418 (2)
O1—C2	1.3674 (18)	C5—C8	1.456 (2)
O1—C1	1.4440 (19)	C6—C7	1.365 (2)
O2—C7	1.3698 (18)	C6—H6	0.9300
O2—C1	1.4417 (18)	C8—C9	1.341 (2)
O3—C10	1.2100 (19)	C8—H8	0.9300
O4—C12	1.2153 (18)	C9—C12	1.482 (2)
O5—C12	1.3316 (18)	C9—C10	1.505 (2)
O5—C13	1.4533 (18)	C10—C11	1.507 (2)
C1—H1A	0.9700	C11—H11A	0.9700
C1—H1B	0.9700	C11—H11B	0.9700
C2—C3	1.370 (2)	C13—C14	1.500 (2)
C2—C7	1.388 (2)	C13—H13A	0.9700
C3—C4	1.400 (2)	C13—H13B	0.9700
C3—H3	0.9300	C14—H14A	0.9600
C4—C5	1.398 (2)	C14—H14B	0.9600
C4—H4	0.9300	C14—H14C	0.9600
			
C2—O1—C1	105.73 (11)	C5—C8—H8	115.0
C7—O2—C1	105.82 (12)	C8—C9—C12	122.92 (14)
C12—O5—C13	115.22 (11)	C8—C9—C10	125.93 (14)
O2—C1—O1	107.25 (12)	C12—C9—C10	111.14 (12)
O2—C1—H1A	110.3	O3—C10—C9	122.29 (14)
O1—C1—H1A	110.3	O3—C10—C11	123.62 (14)
O2—C1—H1B	110.3	C9—C10—C11	114.05 (12)
O1—C1—H1B	110.3	C10—C11—Cl	113.15 (11)
H1A—C1—H1B	108.5	C10—C11—H11A	108.9
O1—C2—C3	127.81 (14)	Cl—C11—H11A	108.9
O1—C2—C7	110.13 (13)	C10—C11—H11B	108.9
C3—C2—C7	122.03 (14)	Cl—C11—H11B	108.9
C2—C3—C4	116.55 (14)	H11A—C11—H11B	107.8
C2—C3—H3	121.7	O4—C12—O5	124.39 (14)
C4—C3—H3	121.7	O4—C12—C9	122.08 (14)
C5—C4—C3	122.29 (14)	O5—C12—C9	113.52 (12)
C5—C4—H4	118.9	O5—C13—C14	107.37 (13)
C3—C4—H4	118.9	O5—C13—H13A	110.2
C4—C5—C6	119.44 (13)	C14—C13—H13A	110.2
C4—C5—C8	117.17 (14)	O5—C13—H13B	110.2
C6—C5—C8	123.38 (13)	C14—C13—H13B	110.2
C7—C6—C5	117.36 (13)	H13A—C13—H13B	108.5
C7—C6—H6	121.3	C13—C14—H14A	109.5
C5—C6—H6	121.3	C13—C14—H14B	109.5
C6—C7—O2	127.85 (14)	H14A—C14—H14B	109.5
C6—C7—C2	122.28 (14)	C13—C14—H14C	109.5
O2—C7—C2	109.85 (13)	H14A—C14—H14C	109.5
C9—C8—C5	129.98 (14)	H14B—C14—H14C	109.5
C9—C8—H8	115.0		
			
C7—O2—C1—O1	10.89 (16)	C3—C2—C7—O2	−177.48 (13)
C2—O1—C1—O2	−10.58 (16)	C4—C5—C8—C9	165.21 (16)
C1—O1—C2—C3	−175.80 (15)	C6—C5—C8—C9	−14.7 (3)
C1—O1—C2—C7	6.33 (16)	C5—C8—C9—C12	179.08 (14)
O1—C2—C3—C4	−178.16 (14)	C5—C8—C9—C10	−2.2 (3)
C7—C2—C3—C4	−0.5 (2)	C8—C9—C10—O3	94.20 (19)
C2—C3—C4—C5	−1.2 (2)	C12—C9—C10—O3	−86.95 (16)
C3—C4—C5—C6	2.5 (2)	C8—C9—C10—C11	−87.96 (19)
C3—C4—C5—C8	−177.38 (14)	C12—C9—C10—C11	90.88 (15)
C4—C5—C6—C7	−2.0 (2)	O3—C10—C11—Cl	−0.83 (19)
C8—C5—C6—C7	177.84 (14)	C9—C10—C11—Cl	−178.63 (10)
C5—C6—C7—O2	178.49 (13)	C13—O5—C12—O4	−5.4 (2)
C5—C6—C7—C2	0.4 (2)	C13—O5—C12—C9	175.26 (13)
C1—O2—C7—C6	174.55 (15)	C8—C9—C12—O4	−175.13 (15)
C1—O2—C7—C2	−7.17 (16)	C10—C9—C12—O4	6.0 (2)
O1—C2—C7—C6	178.93 (14)	C8—C9—C12—O5	4.2 (2)
C3—C2—C7—C6	0.9 (2)	C10—C9—C12—O5	−174.69 (12)
O1—C2—C7—O2	0.53 (17)	C12—O5—C13—C14	−178.22 (13)

### Hydrogen-bond geometry (Å, °)

**Table:**

*D*—H···*A*	*D*—H	H···*A*	*D*···*A*	*D*—H···*A*
C1—H1a···O4^i^	0.97	2.50	3.348 (2)	146
C11—H11b···O3^ii^	0.97	2.36	3.1585 (18)	139

Symmetry codes: (i) −*x*+3/2, −*y*, *z*+1/2; (ii) *x*+1, *y*, *z*.
